# Peripheral human mAb therapy yields modulation of neuroinflammation and long-term functional recovery from lyssavirus infection

**DOI:** 10.1080/22221751.2026.2736987

**Published:** 2026-09-18

**Authors:** Celeste Huaman, Yan Xu, Nathaniel M. Lott, Elise M. Santorella, Madeline Rader, Allison M. Strazzella, Caitlyn Clouse, Jocelyn M. King, Mattie Melnyk, Junping Hong, Lianying Yan, Adrian C. Paskey, Andrea E. Luquette, Francisco Malagon, Regina Z. Cer, Kimberly A. Bishop-Lilly, Daniel P. Perl, Christopher C. Broder, Kai Xu, Brian C. Schaefer

**Affiliations:** a Department of Microbiology and Immunology, Uniformed Services University, Bethesda, MD, USA; b Henry M. Jackson Foundation for the Advancement of Military Medicine, Inc., Rockville, MD, USA; c Department of Veterinary Biosciences, The College of Veterinary Medicine, The Ohio State University, Columbus, OH, USA; d Center for Retrovirus Research, The Ohio State University, Columbus, OH, USA; e Genomics and Bioinformatics Department, Naval Medical Research Command – Frederick, Fort Detrick, MD, USA; f Leidos, Inc., Reston, VA, USA; g DoD/USU Brain Tissue Repository and Neuropathology Program, Uniformed Services University, Bethesda, MD, USA; h Department of Pathology, Uniformed Services University, Bethesda, MD, USA; i Department of Microbial Infection and Immunity, The Ohio State University, Columbus, OH, USA

**Keywords:** Lyssavirus, rabies, monoclonal antibody, cryoelectron microscopy, pre-clinical research, transcriptomics, longitudinal, bioluminescence imaging

## Abstract

Rabies is a fatal encephalitis caused by viruses in the lyssavirus genus. We previously demonstrated that intraperitoneal administration of a single dose of neutralizing human monoclonal antibody (mAb) F11 can protect mice from fatal lyssavirus infection, post-central nervous system (CNS) invasion. However, the molecular basis of F11 neutralization remains unknown. Here, we use structural and functional analyses to define neutralizing activity of F11 and the related mAb, A6. Cryo-electron microscopy (EM) and negative-stain EM reveal that both mAbs bind Domain III of the glycoprotein G, recognizing a prefusion-specific epitope distinct from previously characterized antibodies including RVC20. Binding stabilizes the prefusion state, blocking membrane fusion. We furthermore found that a single dose of either A6 or RVC20 protected animals from mortality induced by Australian bat lyssavirus (ABLV), while promoting substantial long-term functional recovery. Moreover, A6 similarly protected animals from mortality following infection with a currently circulating wild isolate of *rabies virus* (RABV). Transcriptomics analysis of brain RNA and protein-based analysis of brain tissue homogenates demonstrated that therapy with A6 broadly reduced expression of neuroinflammatory mediators during the acute phase of infection. Interestingly, infection of animals with a non-lethal attenuated mutant of ABLV resulted in similar neuroinflammation. Thus, a robust neuroinflammatory response to ABLV does not predict mortality. Overall, peripheral therapy with A6 and similar neutralizing mAbs promotes survival and long-term functional recovery from CNS-resident lyssavirus infection in a manner that includes suppression of the G pre- to postfusion transition and modulation of neuroinflammation.

## Introduction

Viral encephalitis is a severe and often life-threatening infection of the brain caused by neurotropic viruses from numerous distinct families, including flaviviruses, herpesviruses, enteroviruses, paramyxoviruses and rhabdoviruses [1,2]. Once encephalitic viruses reach the central nervous system, therapeutic options are extremely limited, with supportive care being the only option for the vast majority of such infections [3,4]. As emerging and re-emerging neurotropic viruses continue to pose public health threats, the development of effective therapies for viral encephalitis remains an urgent and unmet public health need [5,6].

Lyssaviruses, the deadliest genus of neurotropic viruses, traffic from an exposure site in peripheral tissues to the brain, where they then trigger rabies encephalitis, causing near uniform mortality [7,8]. Despite the availability of effective vaccines and post-exposure prophylaxis (PEP), rabies is responsible for approximately 60,000 deaths annually, disproportionately impacting countries with limited access to prompt clinical care [9-11]. Importantly, once lyssavirus enters the human CNS, triggering symptomatic rabies, there is no known therapy that can prevent mortality [7,8].

Currently, there are 18 known species of lyssaviruses, which are genetically and antigenically segregated into two phylogroups, with three divergent species remaining ungrouped [12]. *Rabies virus* (RABV) and Australian bat lyssavirus (ABLV) are closely related members of phylogroup I of the lyssavirus genus [13]. While RABV transmitted by dog bites is responsible for the majority of human rabies cases worldwide, ABLV is a bat-borne virus that has caused fatalities in 100% of known human cases in Australia [13]. The disease caused by ABLV presents with clinical features in humans that are indistinguishable from classical rabies, with a short prodromal phase characterized by fever and malaise, followed by encephalitic symptoms, including autonomic dysfunction with spasms, hypersalivation, and extreme agitation, ultimately leading to coma and death. Histological findings are also highly similar to classical rabies, with neurons exhibiting cytoplasmic inclusions (Negri bodies) and brain tissue having infiltrates of inflammatory cells, with prominent perivascular cuffing [14-16]. ABLV infection in mice leads to disease signs that are also key hallmarks of RABV infection, including weight loss, lethargy, tremors, uncoordinated gait, and hind-limb paralysis, with 20% weight loss being the disease criterion most often leading to humane euthanasia [17]. Inflammatory infiltrates are present in the brain soon after viral replication commences in this tissue [18].

We have previously described two human monoclonal antibodies (mAbs), F11 and A6, that efficiently neutralize phylogroup I lyssaviruses (including RABV and ABLV) *in vitro* [19]. Using a luciferase (Luc)-based system to longitudinally trace ABLV-Luc transcriptional activity during infection, we previously demonstrated that peripheral administration of a single peripheral dose of F11 efficiently protects animals from morbidity and mortality, even when delivered after robust ABLV transcriptional activity in the CNS. F11 therapy was associated with a significant reduction of the viral load in the spinal cord and brain [18]. In addition, F11 treatment induced substantial immune cell infiltration into the brain and altered the composition of immune cell subsets [18], suggesting that peripheral mAb therapy influences CNS immune dynamics, including expression of genes and soluble mediators. Importantly, F11 therapy was shown to be ineffective in mice lacking an adaptive immune response, with both CD4 T cells and a functionally intact mAb Fc region being required for protection from mortality. Collectively, these results challenge the long-standing tenet that peripherally administered antibodies are ineffective against lyssavirus infection, once CNS penetration has occurred [18,20].

To further reinforce and extend our prior observations regarding mAb therapy for CNS resident lyssavirus infection, we first explored structural features of the interaction between mAbs F11 and A6 and the ABLV surface glycoprotein (G), to better understand the mechanism of neutralization. We then assessed the *in vivo* efficacy of mAbs A6 and RVC20 [21,22], to determine whether the therapeutic efficacy characteristic of F11 [18] extends to other neutralizing antibodies, when delivered peripherally in single-dose format. We also tested the efficacy of A6 in rescue from mortality following infection with Ken-1, a recently isolated wild strain of RABV. Furthermore, we performed transcriptomic analyses of RNA harvested from brains of ABLV-infected animals to define the effect of A6 on infection-associated gene expression changes. Finally, we performed analysis of transcriptomic analysis of gene expression in mice infected with an attenuated strain of ABLV to better understand whether or not the gene expression changes associated with A6 therapy are mechanistically linked to protection from mortality. Overall, through this study, we define mechanisms by which peripherally administered A6 and F11 neutralize lyssavirus, and we demonstrate that both A6 and RVC20, like F11, effectively protect against mortality when administered in single-dose format, with A6 being particularly effective at promoting long-term functional recovery.

## Materials and methods

### ABLV-G Pleckstrin homology domain (PHD) construct design and antigen preparation

The soluble ABLV-G-PHD construct was designed using a homology structural model (Swiss-Model) with a circular permutation linking two discontinuous regions of the domain via a 2×G4S linker. This approach is based on previously validated strategies for stabilizing domain III folds while preserving antigenic epitopes [23]. To confirm proper folding and antigenicity, binding affinity to A6 and F11 Fabs was assessed by biolayer interferometry prior to structural studies. The structural correspondence with the native prefusion G trimer (PDB: 7U9G) was further validated by calculating the Root-Mean-Square Deviation (RMSD) between the PHD in our cryo-EM structure and the corresponding region of the native G structure using the Matchmaker tool in ChimeraX. The construct includes an HRV-3C protease cleavage site and a C-terminal Fc tag to facilitate expression and purification. Expi293 cells were diluted to 1.2 × 10^6^ cells/mL and transfected with 1 mg/L of the ABLV-G-PHD-Fc plasmid using Turbo293 according to the manufacturer’s protocol. After five days, media were clarified by centrifugation at 10,000 × g, filtered, concentrated, and loaded onto Protein A resin (Cytiva) by gravity flow. The resin was washed with three column volumes of HEPES buffer containing 150 mM NaCl, and bound protein was eluted with Glycine pH 3. The Fc tag was removed by HRV-3C protease cleavage, and the protein was further purified by size exclusion chromatography using a HiLoad 16/600 Superdex 200 pg column (Cytiva).

### Antibody preparation and antigen–antibody complex preparation

Antibody RVC20 was synthesized based on the published sequence [23]. Antibodies A6, F11, and RVC20 heavy chain constructs were made by fusing VH-CH1 sequences to a C-terminal hexahistidine (6×His) tag. DNA sequences for heavy and light chains of all antibodies were codon-optimized for mammalian expression, synthesized by GenScript, and cloned into the pVRC8400 plasmid. Heavy and light chain plasmids were co-transfected at a 1:1 mass ratio into Expi293 cells. Antibodies were purified using Ni-NTA resin (Roche), and eluted Fab domains were further purified by size exclusion chromatography using a HiLoad 16/600 Superdex 200 pg column (Cytiva). The ABLV-PHD protein was incubated with a 1.5-fold molar excess of antibody on ice for 1 h to form complexes, which were subsequently purified by size exclusion chromatography as described above.

### Affinity measurement by biolayer interferometry

Binding affinities of ABLV-PHD to A6 and F11 Fabs were measured using a ForteBio Octet RED system. Fc-tagged PHD was captured on Protein A biosensors for 60 s at 50 μg/mL, followed by dipping into Fab at 50 μg/mL. Association and dissociation kinetics were recorded for 60 s each. KD values were calculated using ForteBio Octet Data Analysis software with a 1:1 binding model.

### Negative-stain EM structural analyses and cryo-electron microscopy structural analyses

Binary or ternary antibody-PHD complexes were diluted to 0.02 mg/mL, applied to glow-discharged carbon-coated copper grids, and stained with 2% uranyl acetate. Images were collected at 78,000× magnification on a Tecnai TF20 microscope (200 kV) equipped with a 2 × 2k CMOS camera (pixel size 3.69 Å) using Serial EM. Particle picking, reference-free 2D classification, 3D reconstruction, and refinement were performed in cryoSPARC. Structural analysis and figure generation were performed using PyMOL and Chimera.

Three microliters of complex at 0.4 mg/mL were deposited on 300-mesh 1.2/1.3 carbon grids (Quantifoil) and vitrified using an FEI Vitrobot Mark IV at 4°C, 95% humidity, 30 s wait time, 4 s blot time, and blot force −2. Automated data collection was performed on a Titan Krios microscope using a Gatan K3 detector via Thermo Fisher EPU software in counting mode, with a total dose of 50 e^−^/Å². Data processing was carried out in CryoSPARC. Iterative model building was performed in Coot, followed by real-space refinement in Phenix. Geometry and map fitting were assessed using MolProbity and EMRinger. Data collection and refinement statistics for cryo-electron microscopy are shown in Table S1. Figures were generated using PyMOL and ChimeraX. The ABLV PHD:A6:RVC20 structure has been deposited to the Protein Data Bank with the accession number PDB 38PM.

### Cell lines and viruses

HEK-293 T (ATCC Cat#CRL-3216) was used for the propagation of ABLV-Luc. N2a (Cat#CCL-131) were used for isolation, propagation, and titering of RABV-Ken-1 (wild isolate) and for the titering of ABLV-Luc [17]. Both cell lines were passaged using DMEM (GE Healthcare Life Sciences) supplemented with 10% heat-inactivated fetal bovine serum (FBS) (Sigma Aldrich) and 1% L-glutamine (Corning) at 37°C, 5% CO_2_. The Ken-1 virus was isolated from the brain of a rabid dog [24] using murine neuroblastoma (N2a) cells as described [25], with modifications. Briefly, N2a cells were incubated with clarified brain homogenate in a 50 mL conical tube for 30 min at 37°C, 5% CO_2_. After incubation, the cell-virus suspension was washed with supplemented Dulbecco's modified Eagle medium (DMEM) and transferred to a T75 flask and incubated at 37°C, 5% CO_2_ for 5 days. At the same time, a small fraction (1/500) of the cell-virus suspension was plated on a 96-well plate to be used for the fluorescent antibody test (FAT) three days later. After a positive FAT, the T75 flask containing virus-infected cells was frozen at −80°C for 1–24 h, lysing the infected cells. The flask was defrosted and cell supernatant containing the isolated virus was purified via ultracentrifugation. The passage 1 virus was then titered as described [26]. Isolated virus was further amplified to generate a stock concentrated enough for *in vivo* studies. N2a cells were seeded at a density of 5 × 10^5^ cells/flask in three T75 cm^2^ flasks and infected with isolated virus at a multiplicity of infection (MOI) of 0.01. Seven days post-infection, fresh N2a cells were seeded at a density of 3 × 10^6^ cells/flask in six T175 cm^2^ flasks and incubated overnight. On the same day, the three T75 flasks were placed at −80°C. The following day, flasks were defrosted and the supernatant was clarified by centrifugation (700×g for 15 min). The clarified supernatant was added evenly to six T175 flasks and the same incubation, freeze/thaw and clarification procedures were conducted, followed by ultracentrifugation to purify virus. This passage 3 virus was then titered and frozen in aliquots. All lyssavirus experiments were conducted under BSL-2 precautions, following protocols approved by the Uniformed Services University (USU) institutional biosafety committee (IBC).

The attenuated variant of ABLV-Luc was a spontaneous mutant that arose in a passage 9 preparation of the viral stock. Upon discovery that this prep was non-lethal, the attenuated stock and several lethal stocks were sequenced using methodology described previously [27]. Raw reads from each of the ABLV-Luc stocks plus negative control libraries were processed using MetaDetector v1.0 [28]. In brief, the reads were trimmed using FastQC [29], human host removal was performed, and cleaned reads were assembled using metaSPAdes v3.15.5 [30]. Assemblies were visualized using Bandage v0.8.1 [31], and trimmed reads were mapped back to the contigs using BWA-MEM v0.7.17 [32], deduplicated and sorted using Samtools v1.16.1 [33], then inspected visually using Tablet v1.21.02.08 [34] for mutations, depth, and breadth of coverage. The ABLV-Luc 2015 genome obtained via *de novo* assembly was annotated using Prokka v1.12 [35], and freebayes variant calling tool v1.3.5 [36] was used to systematically check for SNVs > Q20 in all samples as compared to this original stock reference. The analysis was repeated using the sequence of the parent plasmid (pCMV-ABLVp_FL-luc) as a reference, both with freebayes and CLC Workbench. NCBI ORF finder and blastp v2.2.26 were used to identify the amino acid sequence for each ABLV gene from each main contig [37]. The translated sequences were viewed in an alignment using COBALT [38]. Via sequencing analysis of the ABLV passage 9 viral stock, a single point mutation in the G gene (K245E) was identified at a 62% frequency. No other mutations were detected in the passage 9 virus, and no mutations were detected in any of the other analysed stocks.

### Mice, virus challenge and antibody therapy

Mice were 8 (+/- 1) week old female albino C56BL/6 (B6-albino) or C57BL/6 obtained from Jackson Laboratories. For lyssavirus infections, mice (*n* = 3–8 per group) were placed in an isoflurane induction chamber until loss of consciousness. Mice were inoculated with the corresponding dose (ABLV-Luc: 2 × 10^5^ FFU and RABV-Ken-1: 2 × 10^3^, 2 × 10^4^, or 2 × 10^5^ FFU) of virus (or DMEM for mock infections) in a 20 μL volume via footpad infection. For mAb therapy, mice received a single dose of mAb (A6, RVC20, or m102.4) at a dose of 10 mg/kg on day 5, 7, or 9 post-infection. For all mouse infection studies, criteria for humane endpoint euthanasia by ketamine/xylazine overdose was ≥ 20% weight loss. Animals were monitored daily during the first two weeks of an infection experiment, and then three times a week. Mice were assessed for the development of clinical signs of disease, weight change, and motor function (see *Behavior Assay*, below). All animal experiments were performed under ABSL-2 conditions in the USU specific pathogen free animal facility. Experimental protocols were approved by the USU institutional animal care and use committee (IACUC) in accordance with all applicable Federal regulations governing the protection of animals in research.

### Behavior assay

To assess motor coordination, endurance, and muscle strength, a mesh inversion test was performed [39]. Briefly, mice were placed on a wire mesh approximately 30 cm from a padded surface and then inverted. The latency to falling was recorded for up to 60 s. This test was conducted three times per session for each animal, and the average value was quantified and plotted as latency to fall (s) over time (days post-infection).

### In vivo bioluminescence imaging (BLI)

Mice were imaged as described [18]. Briefly, prior to imaging, mice were anesthetized with isoflurane and injected via I.P. with 150 mg/kg of D-luciferin (Gold Biotechnology, catalog #LUCK1G). Following a 10 min rest period, mice were placed in an isoflurane induction chamber before being transferred to the PE IVIS Spectrum CT (Revvity, Inc.) where they were maintained under isoflurane anaesthesia continuously for the duration of image acquisition (1 min maximal duration).

### In vitro neutralization assays

Neutralization assays with ABLV-Luc were performed as previously described [18]. For neutralization assays involving RABV-Ken-1, virus was added to 2-fold serial dilutions of F11, A6, or RVC20 at an MOI of 0.1 and incubated for 90 min at 37°C, 5% CO_2_. After incubation, the virus-mAb mixture was added to N2a cells in a black-walled clear bottom 96-well plate (Corning) in a volume of 0.2 mL supplemented DMEM, followed by incubation for an additional 48 h at 37°C, 5% CO_2_. After incubation, cells were fixed using 4% paraformaldehyde (PAF) for 1 h, followed by staining with anti-rabies-G conjugated to FITC (FUJIREBIO Catalog #800-092) O/N at 4°C. Wells were imaged and fluorescent foci were counted using a CTL S6 analyzer (Cellular Technology Limited). Neutralization percent was calculated by subtracting the FFU of virus-mAb (FFUmAb) wells from the respective well without mAb (FFU0): neutralization (%) = 100 ×     ((FFU0-FFUmAb)/FFU0). Each mAb concentration was tested in duplicate or in triplicate.

### Analysis of soluble mediators

Blood and brain tissue were collected from mice on day 14 post-infection. Prior to collection, mice were euthanized using ketamine (100 mg/kg) and xylazine (10 mg/kg), at a volume of 300 μL/10 g. Blood samples were centrifuged (2,000×g for 10 min at 4°C) after clotting to separate serum, followed by aliquoting. A 20% w/v clarified brain homogenate was prepared using 1× PBS. Serum and clarified brain homogenates were stored at −80°C until ready to use. To inactivate virus, samples were thawed and incubated with 0.008% glutaraldehyde at 4°C for at least 2 h prior to use. Specific soluble analytes were then detected using the LEGENDplex^TM^ Murine Virus response 13-plex (BioLegend catalog #740622), according to the manufacturer’s instructions. Briefly, clarified brain homogenates or serum samples were diluted at 1:2 and incubated with capture beads for 2 h at ambient temperature (RT) while shaking. Samples were then incubated with detection antibodies for 1 h at RT with shaking, followed by biotinylated detection antibody for 30 min RT with shaking. Data were acquired using a Cytek Aurora flow cytometer and analysed using the BioLegend LEGENDplex^TM^ Data Analysis Software Suite. Analyte concentrations were calculated via comparison to internal standards.

### Quantitative real-time PCR (qRT-PCR)

qRT-PCR was performed as previously described [18]. Briefly, total RNA was extracted using Trizol (Invitrogen, Cat# 15596026) and a bead homogenizer (Bullet Blender 5 Gold, Next Advance Laboratory Instruments). We then synthesized cDNA using specific primers to reverse-transcribe positive-sense N transcripts and negative sense genomes in the M-G intergenic region, followed by PCR with the corresponding primers and TaqMan probes. The resulting *C_t_* values were used to calculate transcript numbers per microgram of total RNA via interpolation against a standard curve.

### Total RNA sequencing (RNA-Seq)

For all RNA-seq experiments, total RNA was quantified using the Qubit RNA Broad Range Assay Kit (Thermo Fisher Scientific, cat. #Q10211) and normalized to a concentration of 20 ng/μL. For each sample, 500 ng of total RNA was used as input for sequencing library preparation using the TruSeq Stranded Total RNA prep kit with Ribo-Zero Plus ligation (Illumina, cat. 20040529) and RNA UD Indexes (Illumina, cat. #20091655).

Sequencing libraries were quantified by real-time PCR using the KAPA library quantification kit for Illumina platforms (Roche, cat. #07960255001) on the LightCycler II 480 system (Roche). Library size distribution and quality were assessed using a Fragment Analyzer (Agilent) with the HS NGS kit for Illumina libraries (Agilent, cat. # DNF-474-1000) to confirm the absence of primer dimers and other library artefacts. Libraries were normalized and pooled immediately prior to sequencing. Pooled libraries were loaded at a concentration of 1.8 nM and sequenced using a NovaSeq 6000 (Illumina) with an S4 Reagent Kit v1.5 (200 cycles) (Illumina, cat. #20028313) to generate paired-end reads while targeting >60 million reads per sample.

For read processing, gzipped FASTQ files were uploaded to Google Cloud Platform and processed using the nf-core/rnaseq workflow (v3.12) executed with Nextflow (v24.04.1) running default parameters [40,41]. Raw sequencing reads underwent quality assessment with FastQC (v.0.11.9), followed by adapter trimming and quality filtering using Trim Galore! (v0.6.7) and Cutadapt (v3.4) [42,43]. Additional sequencing quality control metrics were generated using MultiQC (v1.14), which included analysis from bedtools (v2.30.0), dupradar (v1.28.0), samtools (v1.17), picard (v3.0.0), qualimap (v2.2.2-dev), and rseqc (v3.0.1) [33,44–48]. Reads were aligned to the *Mus musculus* reference genome (GRCm38) using STAR (v2.6.1d) [49]. Transcript quantification was performed using Salmon (v1.10.1) and RSEM (v1.3.1), with gene-level count matrices generated for downstream analysis [50,51].

For differential expression analysis, per-sample gene quantification matrices generated by Salmon were imported into R using the tximport package (v1.12.0) [52]. Statistical analysis was performed using DESeq2 (v1.28.0) in R (v4.0.3) [53]. For all RNA-seq experiments, gene expression was modelled using a generalized linear model that included treatment group as a single factor. Principal component analysis (PCA) was used to evaluate sources of variance among samples prior to hypothesis testing. One sample from the ABLV-A6 treated group was identified as an outlier by PC1 variance and hierarchical clustering of per-sample distances and removed from the downstream analysis. All raw sequencing data, including outlier data, are available upon request.

Differential expression was assessed using the Wald test with multiple testing correction performed using the Benjamini-Hochberg procedure. Empirical Bayes adaptive shrinkage was applied using the ashr R package to improve effect size estimation and mitigate false-positive signals [54]. In all cases, genes with an adjusted *p*-value < 0.05 and an absolute log_2_ fold-change greater than 1 were considered differentially expressed. Gene ontology and pathway enrichment analyses were performed using gene set matrices obtained from msigdbr (v7.4.1) and analysed with the clusterProfiler package (v4.0.5) [55,56]. The raw and processed transcriptomic datasets generated in this study were uploaded to the Gene Expression Omnibus under accession number GEO: GSE347495. The corresponding R code used to perform the analysis is available upon request.

## Brain pathology

Mice were euthanized by lethal overdose, as described above. Brains were removed for fixation in 4% phosphate buffered paraformaldehyde at 4°C. The fixed brain was sectioned in the transverse plane into three slabs which were embedded in paraffin. The paraffin blocks were sectioned at 5 µm thickness, mounted on glass slides and stained with haematoxylin and eosin using a Leica automated tissue stainer (ST5020). Using a Leica Aperio AT2 slide scanner, the stained slides were digitized with a 20× objective. The captured image files were viewed with a Leica Aperio ImageScope application. Identified microscopic images were captured as TIFF files using the ImageScope program, and sections were read and evaluated in a blinded manner.

## Results

### Design and validation of an ABLV-G PHD construct for structural analysis of A6 and F11 binding

Two related pan-phylogroup I broadly neutralizing antibodies, A6 and F11, were previously identified by phage-display panning using VSV virions displaying ABLV-G [19]; however, their binding epitopes on G are unknown. Because Domain III, also known as the PHD, is one of the largest exposed antigenic regions on the glycoprotein and a known target of broadly neutralizing antibodies [23], we hypothesized that A6 and F11 recognize epitopes within this domain.

To test this hypothesis, we designed a soluble ABLV-G PHD construct based on a homology model. A circular permutation strategy connected two discontinuous regions via a flexible 2×G4S linker, producing a stable and expressible antigen (Supplemental Figure 1A). The construct was expressed in Expi293 cells as an Fc-fusion protein, purified by Protein A affinity chromatography and further processed by HRV-3C cleavage and size exclusion chromatography to yield pure PHD (Supplemental Figure 1B). To validate that the engineered PHD construct recapitulates the native antigenic structure, we assessed binding to A6 and F11 Fabs by biolayer interferometry, which demonstrated high-affinity interactions (Kd 15 and 4.4 nM, respectively; Supplemental Figure 1C). Furthermore, alignment of the PHD-A6-RVC20 cryo-EM structure with the prefusion G trimer (PDB: 7U9G) revealed a close structural correspondence, with an RMSD of 0.786 Å over all Cα atoms (Figure 2(A)), confirming that the engineered PHD adopts a conformation compatible with the native glycoprotein.

### Negative stain-EM single particle analysis of the PHD-antibody complexes

To map the epitopes recognized by A6 and F11 and to assess overall complex architecture, negative-stain EM (ns-EM) was performed. The ABLV PHD was mixed with either A6 and RVC20 Fabs or A6 and F11 Fabs. Micrographs of the PHD-A6-RVC20 mixture revealed elongated, worm-like particles. 2D classification and 3D reconstruction revealed an extended architecture with two Fab-like densities connected by a central density (Supplemental Figure 2A–C). In contrast, the PHD–A6–F11 mixture did not form worm-like particles, instead showing isolated Fab-like densities or Fab bound to a small globular density. Fitting the PHD-RVC20 crystal structure (PDB: 6TOU) and a Fab model into the density confirmed that the worm-like particles correspond to two Fabs simultaneously bound to PHD. The absence of such density in the PHD-A6-F11 mixture indicates that A6 and F11 recognize overlapping epitopes, whereas A6 and RVC20 bind distinct, non-overlapping sites (Supplemental Figure 2D).

### Cryo-EM structure of the PHD-A6-RVC20 ternary complex

To further understand the binding mechanism of mAb A6, we set out to determine a high-resolution structure by cryo-EM. The relatively small size of the A6-PHD complex (∼60 kDa) complicates particle alignment and high-resolution reconstruction. However, negative-staining electron microscopy (Ns-EM) results indicated that the ternary complex of A6-PHD-RVC20 is stable and well-defined. By leveraging the simultaneous binding of A6 and RVC20, we generated a larger complex (∼110 kDa), which facilitated alignment and high-resolution structure determination.

Representative micrographs, 2D class averages, and reconstruction quality are shown in Supplemental Figure 3A–C, with local density features supporting accurate model building shown in Supplemental Figure 3D. The final reconstruction enabled atomic modelling of the PHD in complex with both antibodies (Figure 1(A and B)). The structure confirms that A6 and RVC20 engage distinct, non-overlapping epitopes, allowing simultaneous binding without steric clash (Figure 1(B)). The RVC20 interface is consistent with prior data [23], whereas A6 binds the opposite face of the PHD, primarily involving the N-terminal region and adjacent β-strands (j and k).

**Figure 1.**
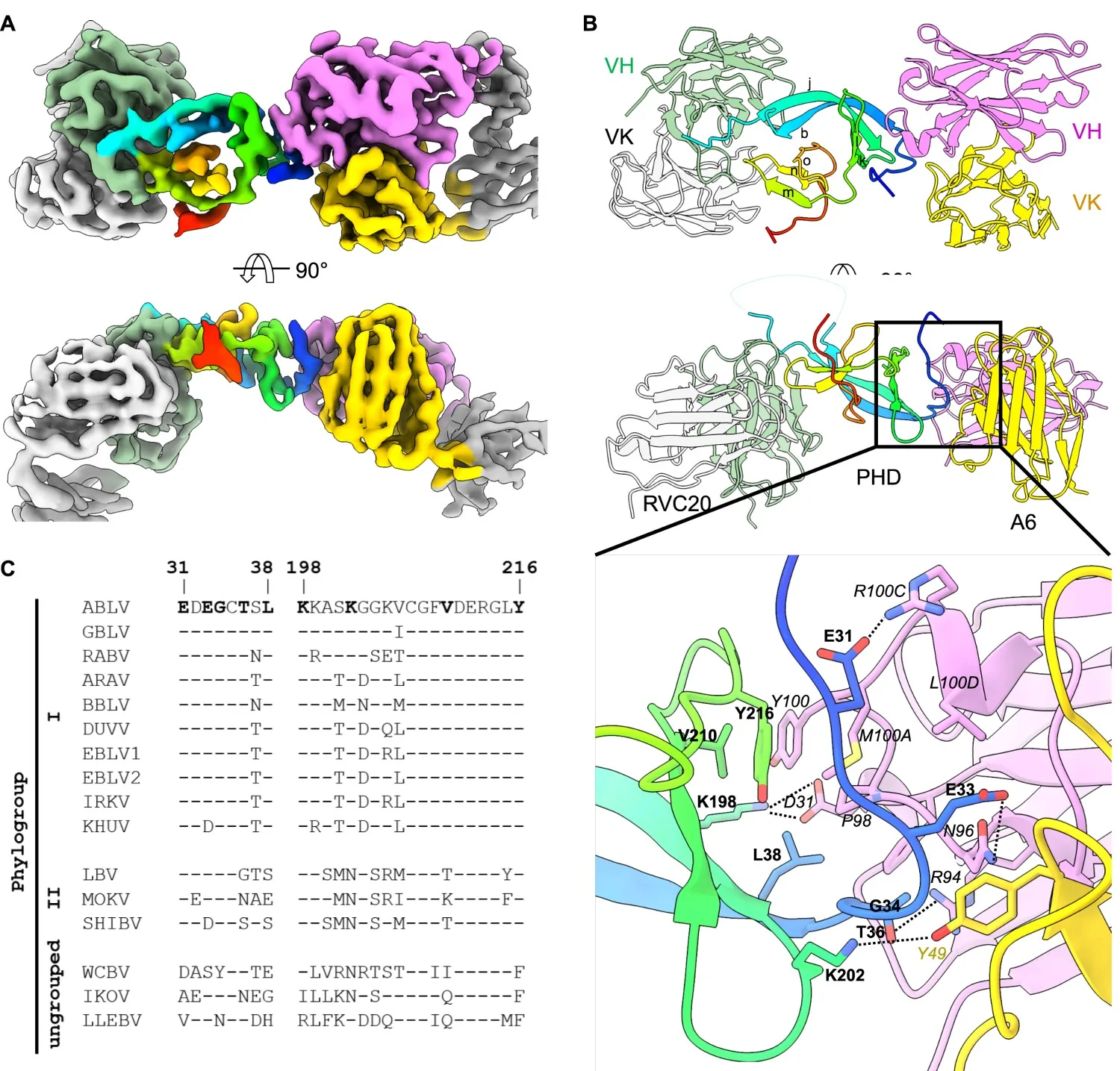
Cryo-EM structure of ABLV-PHD in complex with antibodies A6 and RVC20. (A) Cryo-EM density map of the ternary complex shown in two perpendicular views (top and front), with PHD coloured as a rainbow from blue (N-terminus) to red (C-terminus), A6 (VH magenta, VK yellow), and RVC20 (VH green, VK light grey); (B) Ribbon representation of the complex in the same orientations and colour scheme, with magnified views, below, showing the PHD-A6 interface, where interacting residues are displayed as sticks and labelled (A6 in italic, PHD in bold); (C) Sequence alignment of the A6 epitope across phylogroups, with key residues in bold and residues identical to ABLV indicated by dashes.

### The structural basis of A6 recognition and conservation of the PHD antigenic epitope

The interface between the ABLV PHD and A6 is defined by a network of hydrogen bonds, salt bridges, and hydrophobic interactions, predominantly mediated by the antibody heavy chain (Figure 1(B)). The epitope forms a contiguous surface centred on the N-terminal region of the PHD and adjacent β-strands. For clarity, A6 residues are denoted in *italic* font throughout. A6 engagement is largely driven by residues from heavy chain complementarity-determining region 3 (CDRH3), with additional contributions from CDRH1. Key electrostatic interactions include salt bridges between *R100C* and PHD E31, and between *D31* and PHD K198. These are complemented by multiple hydrogen bonds, including those formed between *N96* and PHD E33, *R94* and PHD T36, and *Y100* and PHD K198. Hydrophobic contacts further stabilize the interface, with *Y100* engaging PHD V210, *M100A* packing against PHD Y216, and *F32* and *P98* interacting with PHD L38. Additional van der Waals interactions are observed between *L100D* and PHD E31/E33, as well as between *F32* and PHD T36, reinforcing local packing at the interface. In contrast, the light chain contributes minimally to antigen recognition, with a single interaction hotspot formed by hydrogen bonds between *Y49* and both the side chain of PHD K202 and the main chain of PHD G34. Together, these interactions define a highly complementary binding interface in which electrostatic specificity and hydrophobic packing jointly stabilize A6 engagement with the PHD (Figure 1(B)).

Sequence alignment across lyssavirus phylogroups revealed that all of the important residues on the ABLV PHD that are involved in A6 recognition are conserved and identical within phylogroup 1 lyssaviruses (Figure 1(C)), except for Khujand virus (KHUV). However, the conservative E33D substitution in KHUV likely preserves a similar interaction capacity. This is consistent with the observed pan-phylogroup 1 neutralization [19]. By contrast, sequence variability at these positions in phylogroups II and the ungrouped lyssaviruses likely disrupts critical contacts, providing a structural explanation for the restricted breadth of A6 and F11 neutralization efficacy [19].

### A6 and RVC20 are specific for the prefusion conformation of RABV G

To understand antibody accessibility in the context of the full glycoprotein, the PHD-A6-RVC20 structure was aligned with both prefusion and postfusion RABV G conformations [57-59]. In the prefusion trimer, the PHD epitope is fully exposed, and both A6 and RVC20 can be accommodated without steric hindrance (Figure 2(A)), demonstrating clear compatibility with the native glycoprotein on the viral surface. By contrast, in silico alignment with the postfusion conformation reveals pronounced steric clashes between the bound antibodies and the central domain (CD) of the glycoprotein (Figure 2(B)). These clashes preclude antibody engagement, indicating that the epitopes recognized by A6 and RVC20 are structurally inaccessible in the postfusion state. These findings establish that A6 and RVC20 are restricted to the prefusion state and excluded from the postfusion conformation. This strict conformational specificity suggests a neutralization mechanism in which antibody binding locks RABV G in its metastable prefusion state, thereby blocking the structural rearrangements required for membrane fusion.

**Figure 2.**
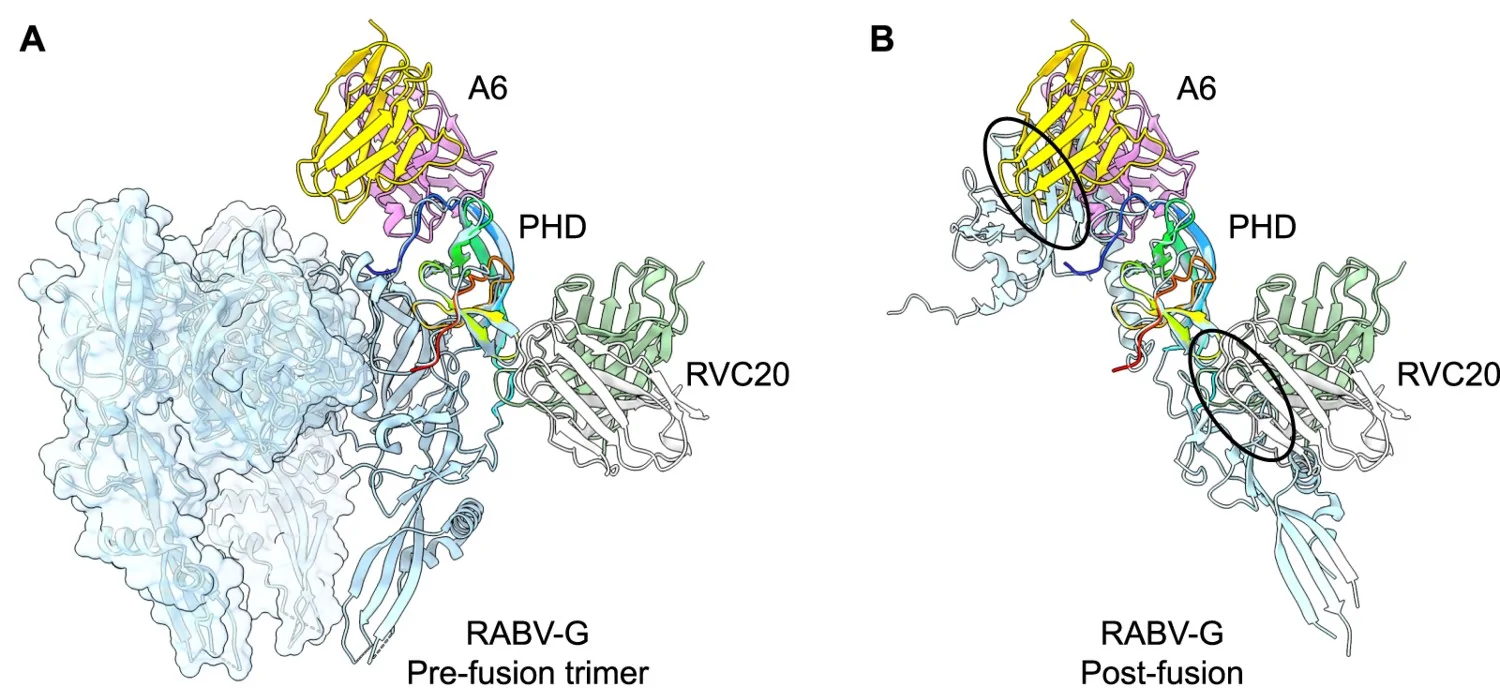
Structural analysis of antibody recognition compatibility in the context of the RABV G ectodomain. (A) The PHD–A6–RVC20 ternary complex aligned to the prefusion G trimer (PDB ID: 7U9G), with the G trimer in light blue, one protomer shown as ribbons and the other two as ribbons within a transparent surface, illustrating A6 and RVC20 binding to PHD within the intact ectodomain; (B) The postfusion G (PDB ID: 6LGW) aligned to panel A via the ternary complex, showing PHD binding in the postfusion conformation and regions of steric hindrance by A6 and RVC20 circled.

### Single-dose therapy with mAbs A6 or RVC20 prevents mortality when administered during or immediately following viral penetrance of the CNS

Our previous *in vitro* studies demonstrated that hmAb A6 neutralizes ABLV with a potency that is essentially indistinguishable from hmAb F11 [17]. To assess whether or not the *in vivo* efficacy of A6 on established ABLV-Luc infection was also similar to what we reported for F11 [18], C57BL/6 albino mice (B6-albino) were infected with 2 × 10^5^ FFU (lethal dose) of ABLV-Luc and treated with a single dose of A6 (10 mg/kg) on day 5, 7, or 9 post-infection (Figure 3(A)). BLI revealed that ABLV-Luc-infected mice treated with A6 on days 5 and 7 post-infection generally exhibited reduced levels of viral transcription in the spinal cord and brain, compared to both day 9-treated ABLV-infected mice and untreated ABLV-Luc-infected controls. Moreover, by day 40 post-infection, there was no detectable viral replication (by BLI) in the spinal cord or brain of A6-treated surviving mice on days 5, 7 or 9 (Figure 3(B,C)). Mice from all treatment groups began to develop clinical signs of disease by day 9 post-infection, similar to infected plus untreated mice. Clinical disease signs and weight loss were fully resolved in the majority of surviving mice. In addition, grip strength, as assessed by a latency to fall assay (see *Materials and Methods*), recovered to near baseline levels in the majority of surviving animals. By contrast, infected untreated mice and A6-treated mice that reached euthanasia criteria exhibited very poor performance in this assay by the time of euthanasia (Figure 3(D)). Finally, despite clear viral replication in the spinal cords and brains of all infected animals, 100% of mice treated on day 5, 33% treated on day 7, and 16% treated on day 9 post-infection survived through the end of the experiment (day 90 post-infection). By contrast, all untreated animals reached euthanasia criteria by day 15 post-infection (Figure 3(E)). Collectively, the data in Figure 3 indicate that A6 therapy leads to attenuation of viral replication in the CNS, substantial functional recovery, and protection from mortality, particularly when administered at day 5 post-infection (*p* < 0.001 vs. no treatment). The kinetics of BLI signal development and the subsequent appearance of clinical signs by day 7 post-infection indicate that day 5 mAb administration occurs during or immediately following viral CNS invasion, consistent with our prior observations of detectable BLI signal in the spinal cords of some animals by day 5 post-infection (as well as the fact that BLI is only able to detect in vivo infection once considerable viral transcription, in excess of 10^4^ N-transcripts/μg of total RNA, has occurred) [17,18].

**Figure 3.**
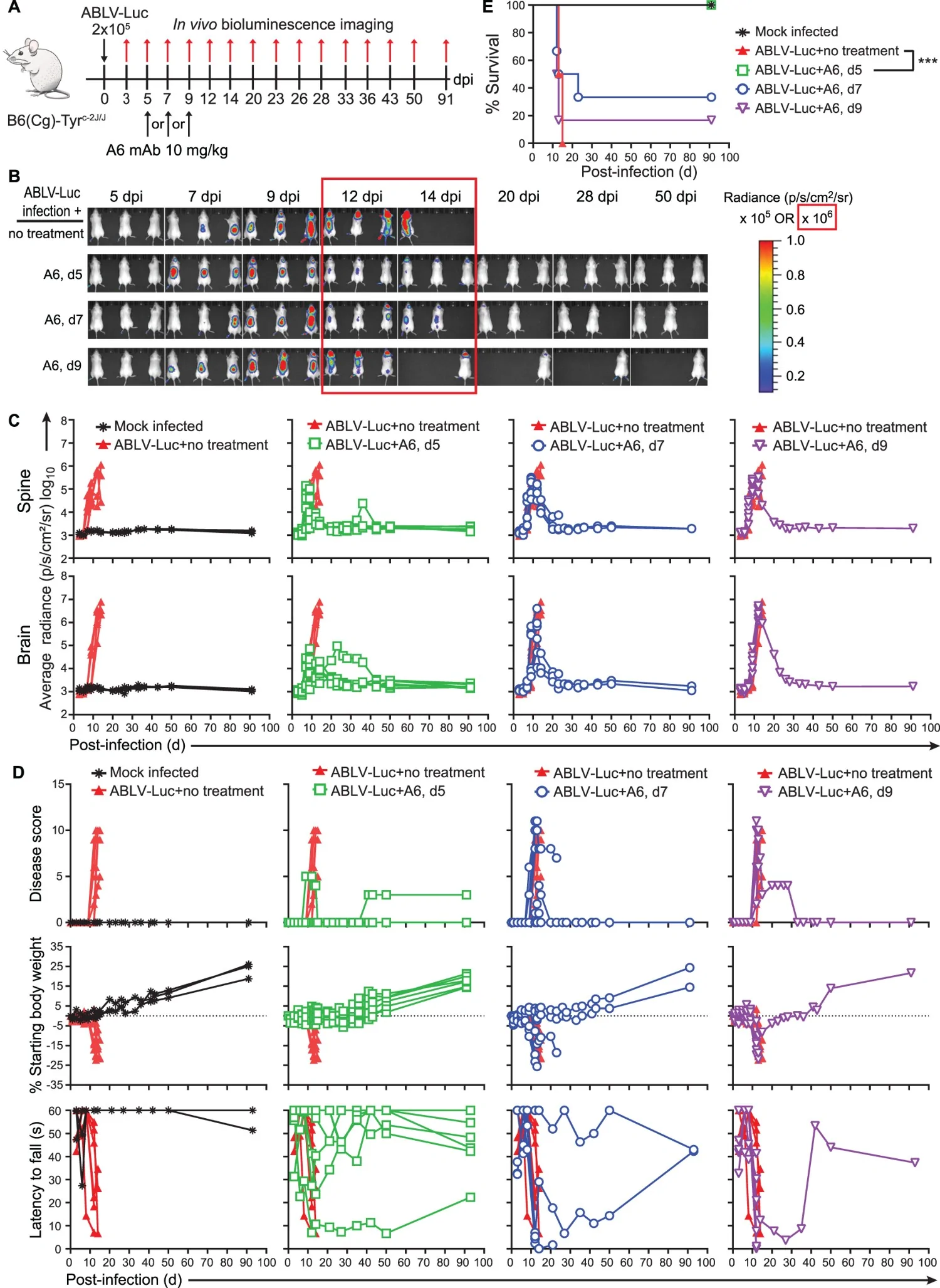
Single-dose therapy with human mAb A6 reduces viral load in the CNS, reverses disease signs, drives motor function improvement and protects animals from mortality. (A) B6-albino mice were uninfected (*n* = 3/group) or infected with ABLV-Luc and were untreated or treated with human mAb A6 (10 mg/kg) in a single dose at day 5, day 7 or day 9 post-infection (*n* = 6/timepoint). In vivo BLI was performed at the indicated timepoints between days 3 and 91 post-infection. (B) BLI images of three representative animals per treatment group at selected time points, showing the magnitude of viral transcription on a pseudocolour intensity scale overlaid on an image of each animal. (C) Quantification of BLI (viral transcription) in the spinal cords (top) and brains (bottom) of individual animals over time, expressed as average radiance. (D) Quantification over time of disease score (top row), percent starting body weight (middle row), and latency to fall (mesh inversion test; bottom row) over time, with each group compared to mice infected with ABLV-Luc in the absence of treatment. (E) Kaplan-Meier plot of survival over time, showing significantly increased survival of ABLV-Luc-infected animals treated at d5 post-infection, vs. ABLV-Luc-infected untreated animals. ***p < 0.001.

Because our structural studies demonstrated that hmAb A6 binds to a site on ABLV G distinct from human mAb RVC20 (Figures 1 and 2), we conducted an experiment to test whether or not RVC20 is also effective as a single-dose therapy for ABLV infection. (RVC20 has previously been shown to be a highly effective therapy for symptomatic rabies when administered as a combination therapy with RVC58, via delivery by a combination of continuous intracerebroventricular infusion and intramuscular injections [21]). We first performed an *in vitro* neutralization assay, comparing RVC20 to F11 and A6. Although neutralization of ABLV was highly efficient for all three mAbs, F11 and A6 were approximately 5.5-fold and 11.5-fold more potent (respectively) than RVC20 (Supplemental Figure 4). We then infected mice with ABLV (2 × 10^5^ FFU) followed by treatment with RVC20 (10 mg/kg) on day 5, 7 or 9 post-infection. Control animals were infected with the same dose of ABLV and given the irrelevant human mAb, m102.4, on day 5 post-infection (Supplemental Figure 5A). BLI data revealed that RVC20 was generally effective as a single-dose therapy, with maximal BLI signals in the spinal cord and brain generally lower in the RVC20-treated animals, as compared to the control group treated with m102.4 (Supplemental Figure 5B-C). Disease score and weight loss data were very similar to observations from animals treated with A6, with most RVC20-treated animals showing return to baseline disease score and recovery of starting weight. RVC20 treatment also yielded some recovery of performance in the latency to fall assay (Supplemental Figure 5D). Notably, however, by the end of the experiment, a higher proportion of surviving RVC20-treated animals exhibited failure to return to a 0 disease score (8/13 = 62%) and a latency to fall >30 s (7/13 = 54%), as compared to the A6-treated group (1/9 = 11% for both measures of functional recovery). Also, the maximal survival effect of RVC20 therapy was observed at day 7 post-infection, with 100% of RVC20-treated animals surviving (*p* < 0.01 vs. m102.4 treatment), whereas 50% of day 5-treated animals and 67% of day 9-treated animals survived (Supplemental Figure 5E). This kinetic is distinct from what was observed for either A6 therapy (Figure 3(E)) or F11 therapy [18], which both exhibited a maximal therapeutic effect when administered day 5 post-infection. Overall, however, our data demonstrated that RVC20 is effective as a single-dose therapy, particularly with respect to preventing mortality at later time points (days 7 and 9) post-infection.

### Single-dose administration of mAb A6 is highly effective against a currently circulating strain of canine RABV

Our previous *in vitro* neutralization data demonstrated that both F11 and A6 are broadly effective against phylogroup I lyssaviruses [19]. Similar data have been published for RVC20 [22]. To compare the efficacy of these three mAbs against a currently circulating canine RABV strain, we performed *in vitro* neutralization assays using the Ken-1 strain (see *Materials and Methods* and [24]). All three mAbs were highly effective for *in vitro* neutralization of Ken-1, with F11 and A6 being approximately 3-fold more potent than RVC20 (Figure 4(A)). We next performed an experiment to test whether or not A6 can effectively neutralize Ken-1 *in vivo* (Supplemental Figure 6A). Initially, mice were challenged with 2 × 10^3^, 2 × 10^4^, or 2 × 10^5^ FFU Ken-1 (*n* = 3 per group) and monitored for the development of clinical disease signs and evaluated for motor function. Interestingly, animals began to develop disease signs as early as day 4 post-infection, strongly suggesting that virus had reached the CNS. Weight loss was observed at day 8 post-infection or later, with rapid weight loss following initial detection (Supplemental Figure 6B). Animals challenged with a dose of 2 × 10^5^ FFU reached humane endpoint criteria and were euthanized between days 10–12 post-infection. Mice in the 2 × 10^4^ FFU dose group followed closely, with animals reaching euthanasia criteria on days 12–13 post-infection. Mice challenged with 2 × 10^3^ FFU did not reach humane endpoint criteria until day 15 and 17 post-infection and one animal survived until the experiment endpoint at day 32 post-infection (Supplemental Figure 6C).

**Figure 4.**
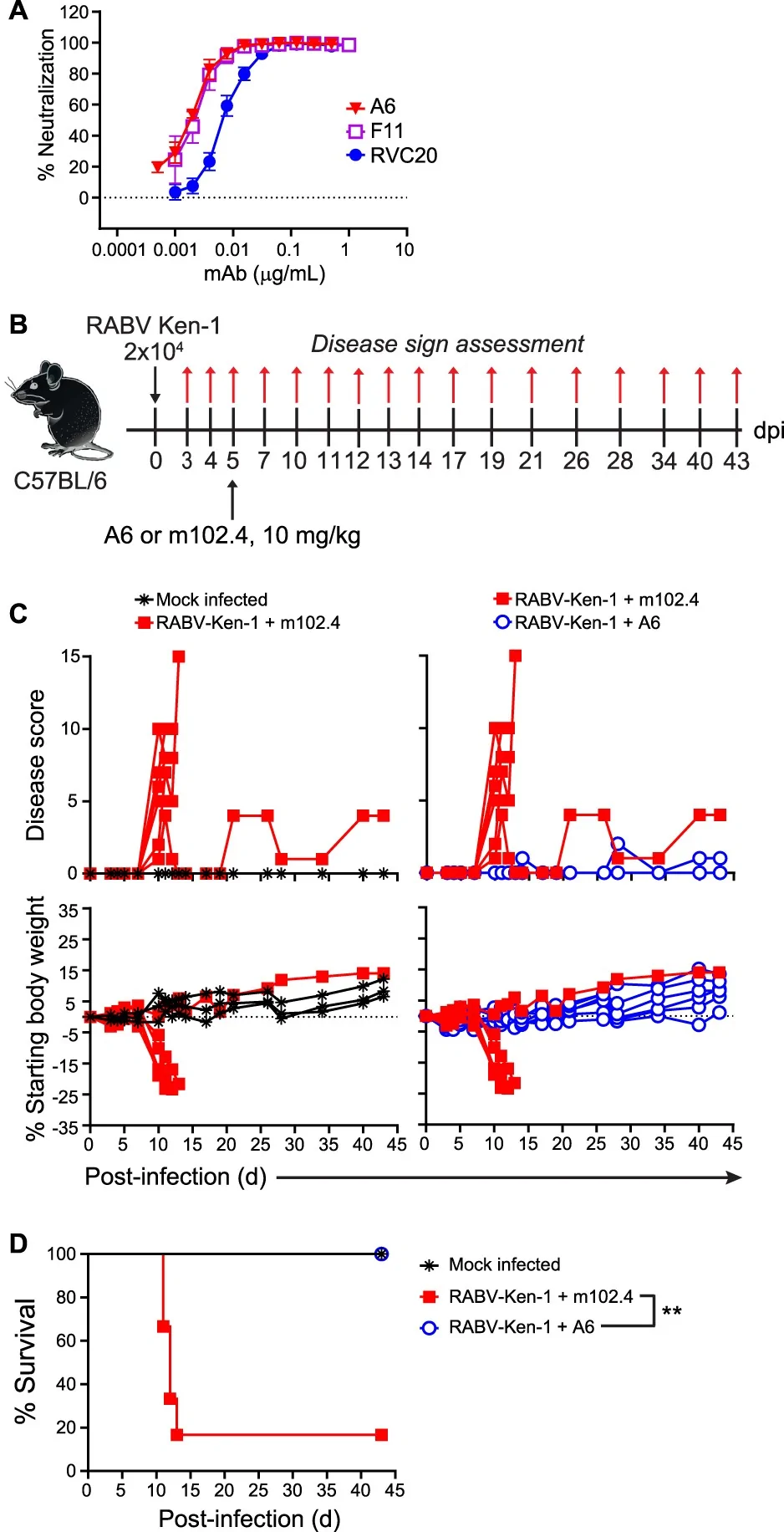
Human mAb A6 is effective *in vitro* and *in vivo* against a currently circulating strain of canine RABV. (A) *In vitro* neutralization of RABV Ken-1 virus with human mAbs A6, F11 and RVC20, with mAb concentration (μg/mL) on the x-axis vs. percent neutralization on the y-axis. The data represent two independent experiments performed in duplicate. Nonlinear regression was used to determine IC_50_ values. Mean values of the calculated IC_50_ ranges were A6, 0.0022 μg/mL; F11, 0.0024 μg/mL; RVC20, 0.0068 μg/mL. (B) C57BL/6 mice were mock infected (*n* = 3) or infected with 2 × 10^4^ FFU Ken-1 virus, followed by administration of A6 (10 mg/kg) or m102.4 (10 mg/kg) on day 5 post-infection (*n* = 6/treatment group). Assessment of disease signs was on the indicated days. (C) Disease score (top row) and percent starting body weight (bottom row) plotted vs. days post-infection. The Ken-1 + m102.4 (control mAb) treatment group is compared individually to the mock infected and Ken-1 + A6 groups. (D) Kaplan-Meier plot of survival over time, showing significantly increased survival of Ken-1-infected animals treated with A6 vs. m102.4. **p < 0.01.

Based on the above findings, we proceeded with 2 × 10^4^ FFU dose of Ken-1 to test the *in vivo* therapeutic efficacy of A6. Infected mice were treated with either A6 or the irrelevant control, m102.4 (each at 10 mg/kg) on day 5 post-infection (Figure 4(B)). As expected, all mice that received m102.4 exhibited disease signs and weight loss by day 10 post-infection. By contrast, animals that were treated with A6 exhibited little to no weight loss and had disease scores ≤ 2 (Figure 4(C)). By the end of the experiment, 100% of mice treated with A6 survived, compared to 18% survival in the m102.4-treated group (*p* < 0.01) (Figure 4D). Collectively, these data demonstrate that A6 is highly effective against a currently circulating canine RABV strain.

### Single-dose administration of mAb A6 reduces inflammatory and antiviral gene and protein expression to levels similar to uninfected controls

To further determine how A6 alters the biology of *in vivo* infection with ABLV-Luc, we performed an experiment in which animals were uninfected or infected with ABLV-Luc. Infected animals were treated either with the control antibody m102.4 or with A6 (each at 10 mg/kg) on day 5 post-infection. Progression of viral infection was monitored by bioluminescence imaging, and disease signs and body weight were also measured until the time of tissue harvest (Supplemental Figure 7A-C). These data were qualitatively and quantitatively similar to the progression of disease observed in the experiment in Figure 3 over the first 13 days post-infection. Additionally, pathology analysis was performed on a similar group of animals. Brains were harvested from uninfected (*n* = 2), ABLV-luc-infected, m102.4-treated (*n* = 3) and ABLV-luc infected, A6-treated (*n* = 3) mice following euthanasia at days 12–13 post-infection. Brains were then fixed, embedded, stained with haematoxylin and eosin, and analysed for pathological findings in a blinded manner. Consistent with other analyses of brain pathology following infection of mice with wild lyssavirus strains [60,61], the only pathological finding was perivascular inflammatory infiltrates in the brains of ABLV-Luc-infected animals, with 3 of 3 of the m102.4-treated animals exhibiting such infiltrates, whereas only 1 of 3 of the A6-treated animals had perivascular infiltrates (Supplemental Figure 8). Thus, the pathology data are consistent with the overall findings in this manuscript, showing that A6 therapy reduces or prevents the occurrence of hallmarks of rabies disease following infection with ABLV-Luc.

For the transcriptomics analysis of animals assessed in Supplemental Figure 7A-C, mice were euthanized at day 14 post-infection, and brains were harvested for extraction of total RNA, followed by a transcriptomics (RNA-Seq) analysis to assess global gene expression levels (see *Materials and Methods*). PCA of normalized RNA-Seq expression profiles revealed separation by treatment group, with samples from m102.4-treated ABLV-infected animals clustering independently from mock-infected controls and ABLV-infected animals treated with A6. Notably, samples from A6-treated ABLV-infected animals clustered closely with mock-infected controls, indicating that treatment with A6 largely mitigated infection-associated transcriptomic gene signatures in whole-brains from infected animals by day 14 post-infection (Figure 5(A)). The transcriptional changes in m102.4-treated ABLV-Luc-infected animals vs. mock-infected controls were largely mediated by inflammatory and antiviral (e.g. interferon-induced) responses, and most of these genes exhibited increased expression in the infected group. By contrast, A6-treated ABLV-infected animals exhibited few differentially expressed genes relative to mock-infected controls, demonstrating that A6 treatment either quickly reversed or entirely negated transcriptome-wide changes to inflammatory and antiviral gene expression profiles in the brains of infected animals. This finding was further emphasized by the fact that comparison of m102.4- to A6-treated animals yielded similar transcriptomic changes to those observed between mock-infected and m102.4-treated animals (Figure 5(B)).

**Figure 5.**
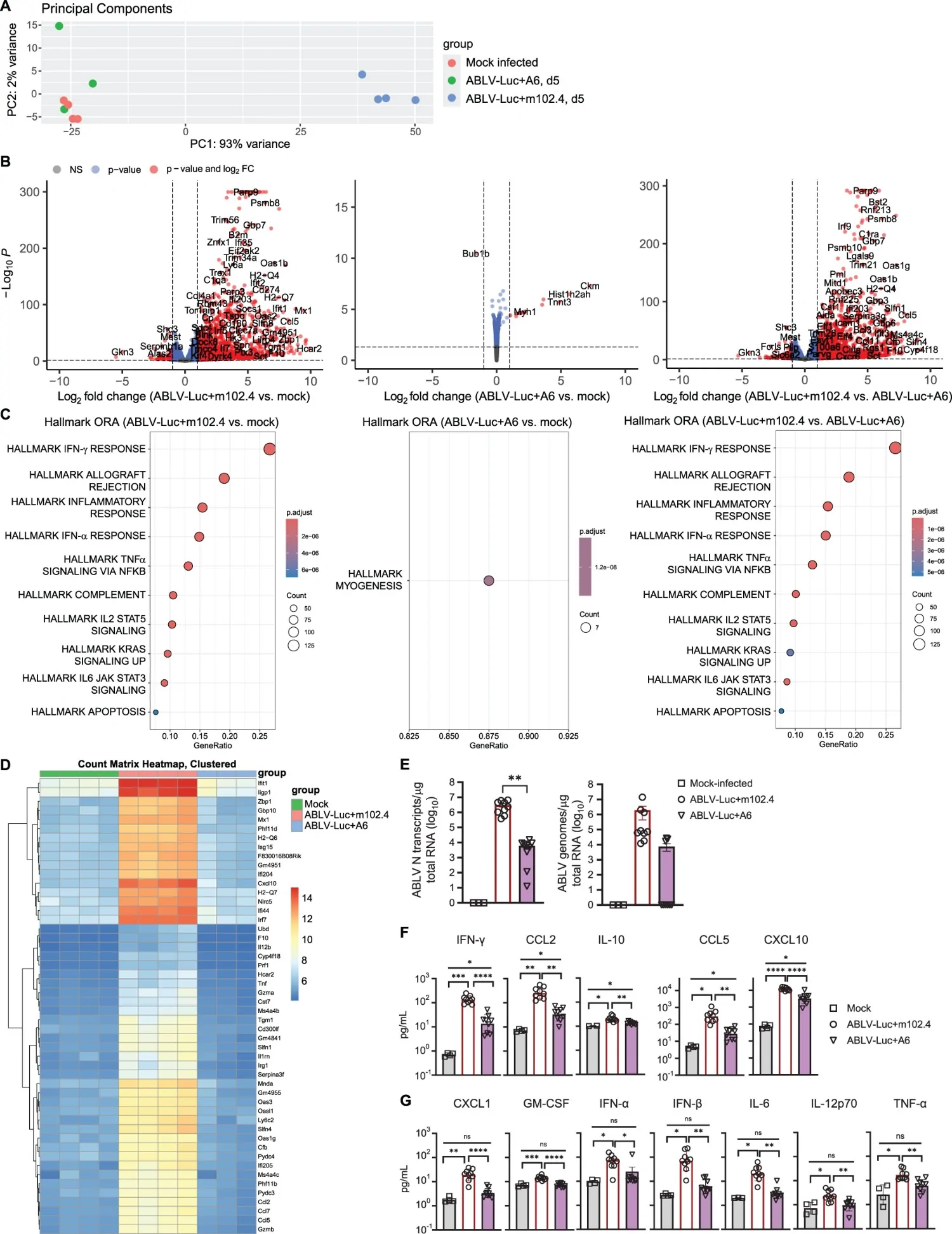
ABLV-Luc infection induces an inflammatory and antiviral response that is largely abrogated by A6 mAb therapy. (A to D) B6 albino mice were mock infected or infected with ABLV-Luc and treated with m102.4 or A6 (10 mg/kg, day 5 post-infection). Total RNA was prepared from brains at 14 days post-infection (*n* = 4/group). (A) Principal component analysis of whole-brain RNA-seq profiles showing clustering of mock-infected and ABLV + A6–treated samples and distinct separation of ABLV + m102.4–treated animals. (B) Volcano plots of DEGs for the comparisons of ABLV + m102.4 vs. mock-infected, ABLV + A6 vs. mock-infected, and ABLV + m102.4 vs. ABLV + A6. Significant DEGs (*p* *<* 0.05) with a log_2_foldchange > 1 are highlighted in red and labelled with gene symbols. Genes below the log_2_foldchange cutoff are coloured in blue. (C) GSEA dot plots showing pathway enrichment for the comparisons ABLV + m102.4 vs. mock-infected, ABLV + A6 vs. mock-infected, and ABLV + m102.4 vs. ABLV + A6. Dot colour indicates the adjusted *p*-value for each enriched pathway, and dot size represents the number of genes contributing to the enrichment within each gene set. (D) Clustered heatmap of the top 50 DEGs from rlog-transformed RNA-seq counts. DEGs were filtered for immune relevance using msigdbr collections. Genes were further selected for a log_2_ fold-change > 1 and base mean >80. Colour intensity indicates relative normalized expression across all samples. (E) qRT-PCR analysis of ABLV-Luc N sense transcripts and antisense genomes in the brains of mice from an infection and mAb treatment experiment performed as in (A-D). Brains were processed for total RNA on day 14 post-infection. Values are plotted as molecules per μg of total brain RNA (*n* = 4 mock-infected and *n* = 9/treatment group). (F and G) Quantification of levels of soluble inflammatory and antiviral mediators in brain homogenates of the same animals assessed in (E). Brains were harvested on day 14 post-infection (*n* = 4 mock-infected and *n* = 9/treatment group). (F) Analytes shown exhibited significant differences between mock infected vs. ABLV-Luc + A6. (G) Analytes shown exhibited no significant differences between mock infected vs. ABLV-Luc + A6. *p < 0.05; **p < 0.01; ***p < 0.001; ****p < 0.00001.

Via gene set enrichment analysis (GSEA), we identified significant enrichment of inflammatory and antiviral pathways in ABLV-infected animals treated with m102.4, relative to both mock-infected mice and ABLV-infected animals treated with A6. GSEA comparing A6-treated ABLV-infected animals to mock-infected controls identified enrichment of only a small number of genes associated with myogenesis (Figure 5(C)). Further reinforcing the above findings, hierarchical clustering of the top 50 differentially expressed genes showed that the identified genes were predominantly associated with inflammation and antiviral responses, including mediators of interferon responses (e.g. IFIT1 (Interferon-Induced Protein with Tetratricopeptide Repeats 1), ZBP1 (Z-DNA-binding protein 1), MX-1 (MX Dynamin Like GTPase 1), ISG15 (Interferon-Stimulated Gene 15)), chemokines (e.g. CXCL10 (C-X-C motif chemokine ligand 10), CCL2 (Monocyte Chemoattractant Protein-1), CCL5 (C–C motif chemokine ligand 5)) and a variety of inflammatory mediators (e.g. TNF (Tumor Necrosis Factor-α), IL12B (Interleukin-12 subunit-β), CFB (Complement Factor B)). These genes were upregulated in m102.4-treated ABLV-Luc-infected animals relative to both mock-infected animals and A6-treated ABLV-Luc-infected animals (Figure 5(D)).

To assess the degree to which the above gene expression changes were reflected in translated gene products, we assessed (in a separate experiment utilizing the same conditions) the levels of 13 antiviral mediators in both brain tissue homogenates and in blood serum at 14 days post-infection. We first assessed infection levels in the brain at the time of euthanasia by qRT-PCR. Similar to our prior assessment of animals treated with F11 at day 7 post-infection [18], qRT-PCR demonstrated substantial reduction in both sense ABLV-Luc N-transcripts and antisense genomes in the A6-treated group (*p* < 0.01 for *N*-transcripts, m102.4 vs A6 treatment) (Figure 5(E)). Analysis of protein levels demonstrated that five mediators (IFN-γ, CCL2, IL-10, CCL5 and CXCL10) were detected at significantly higher levels in the brains of ABLV-Luc-infected animals treated with m102.4 or A6 vs. mock-infected controls. However, in each case, the detected levels of mediators were also significantly greater in m102.4-treated animals vs. A6-treated animals (Figure 5(F)). For seven additional soluble mediators (CXCL1, GM-CSF, IFN-α, IFN-β, IL-6, IL-12p70 and TNF-α), the levels detected in ABLV-Luc-infected and m102.4-treated animals were significantly higher than both mock-infected animals and ABLV-Luc-infected and A6-treated animals. For this group, no significant difference was detected between mock-infected animals and the A6-treated ABLV-Luc-infected group (Figure 5(G)).

By contrast, analysis of these same analytes in the blood serum revealed significant changes in only five of the above analytes (IFN-α, IFN-γ, TNF-α, IL-10 and CXCL10). For each of these analytes, significant differences were detected between m102.4-treated ABLV-Luc-infected and mock-infected animals. Only IFN-α and TNF-α yielded significant differences between m102.4-treated and A6-treated ABLV-Luc-infected animals, and only IL-10 and TNF-α yielded significant differences between mock-infected and A6-treated ABLV-Luc-infected animals (Supplemental Figure 9). Thus, changes in expression of soluble antiviral mediators induced by ABLV-Luc infection are predominantly observed in brain tissue, with a lesser effect in blood serum; changes in protein levels of the above antiviral mediators are reduced or completely abrogated by A6 therapy. Taken together, the data in Figure 5 demonstrate dramatic gene and protein expression changes induced in the brain by infection with ABLV-Luc, with the majority of these changes reflecting inflammatory and antiviral processes. A6 therapy largely abrogates or reverses these changes in gene and protein expression by day 14 post-infection.

### Infection with an attenuated variant of ABLV leads to reduced infection of the brain and reduced induction of inflammatory and antiviral genes

The transcriptomics data in Figure 5 show that A6 therapy results in a dramatic reversal of inflammatory and anti-viral responses induced by ABLV-Luc infection. However, such changes in gene expression may or may not be required for the therapeutic effect of A6. To further address whether or not abrogation of inflammatory and antiviral gene expression is required to protect animals from lyssavirus-induced mortality, we performed experiments with a spontaneously arising attenuated variant of ABLV, which has a single point mutation in the glycoprotein gene (G), K245E (see *Materials and Methods*). Notably, several studies of distinct single point mutants in RABV G have also noted attenuation of pathogenicity, with each mutation involving alteration of charge. Specifically, the E333R [62], D255G [63] and G349E mutations [64] have each been reported to attenuate viral pathogenesis, with various attributed mechanisms.

Infection of two distinct groups of animals infected with ABLV-Luc-WT was compared to animals infected with ABLV-Luc-G(K245E) over a 21-day time course (Figure 6(A)). Although *in vivo* infection with ABLV-Luc-WT and ABLV-Luc-G(K245E) followed similar anatomical progression and kinetics, the intensity of viral transcription in the spinal cord and brain, the manifestation of disease signs and the magnitude of weight loss were reduced for the G(K245E) mutant (Figure 6(B–D)). Importantly, all of the G(K245E) animals survived, whereas 0/6 animals in the WT(A) group and 1/6 animals in the WT(B) group survived (*p* < 0.001 for G(K245E) vs WT(A); *p* < 0.01 for G(K245E) vs WT(B)) (Figure 6(E)). Thus, the G(K245E) mutant is a non-lethal variant of ABLV.

**Figure 6.**
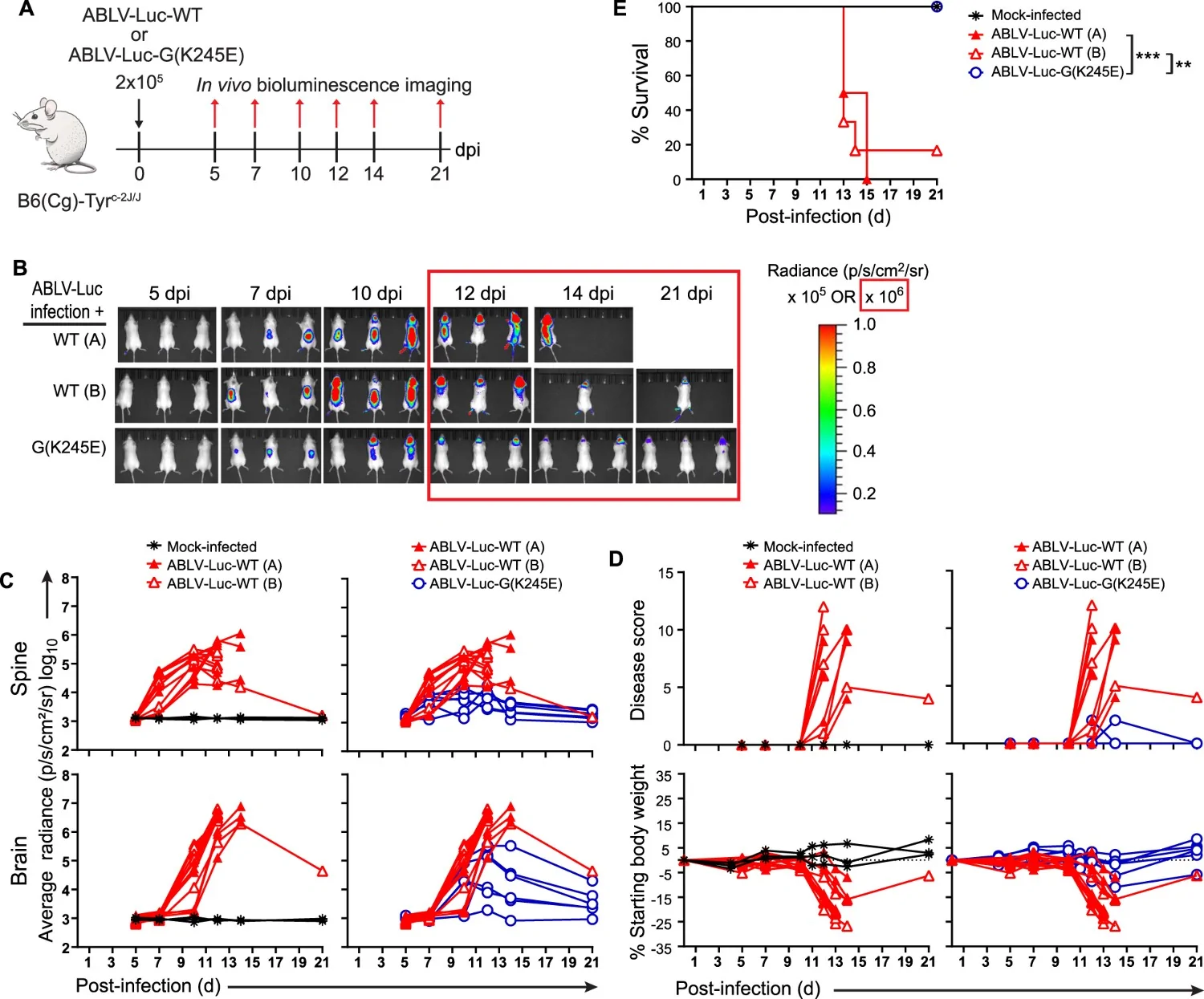
The ABLV-Luc-G(K245E) mutant infects the CNS without causing lethality. (A) B6-albino mice were mock-infected (*n* = 3/group) or infected with either ABLV-Luc-WT or ABLV-Luc-G(K245E) at 2 × 10^5^/animal (*n* = 6/group). Note that WT(A) and WT(B) are two distinct groups of 6 animals infected with ABLV-Luc-WT. *In vivo* BLI was conducted at the indicated timepoints over 21 days post-infection. (B) BLI images of three representative animals per treatment group at selected time points, showing the magnitude of viral transcription on a pseudocolour intensity scale overlaid on an image of each animal. (C) Quantification of viral transcription in the spinal cords (top) and brains (bottom) of individual animals over time, expressed as average radiance. (D) Disease score (top row) and percent starting body weight (bottom row) plotted vs. days post-infection.

To assess gene expression differences between the WT and G(K245E) viruses, we next performed total RNA-seq, comparing those two genotypes to uninfected controls at day 14 post-infection. PCA of normalized RNA-seq expression profiles revealed a clear separation of samples according to infection status. Samples from animals infected with ABLV-Luc-WT and ABLV-Luc-G(K245E) clustered independently from mock-infected controls, indicating that viral infection is the most likely driver of the 88% variance of PC1 (Figure 7(A)). Additionally, RNA-seq analysis demonstrated robust transcriptional changes in both infected groups relative to mock-infected controls. Importantly, infection with ABLV-Luc-G(K245E) produced a transcriptional profile very similar to ABLV-WT, with respect to expression of antiviral and inflammatory genes. Moreover, comparison of ABLV-Luc-WT and ABLV-Luc-G(K245E) revealed modest gene expression changes relative to comparisons of each genotype to the mock-infected group (Figure 7(B)).

**Figure 7.**
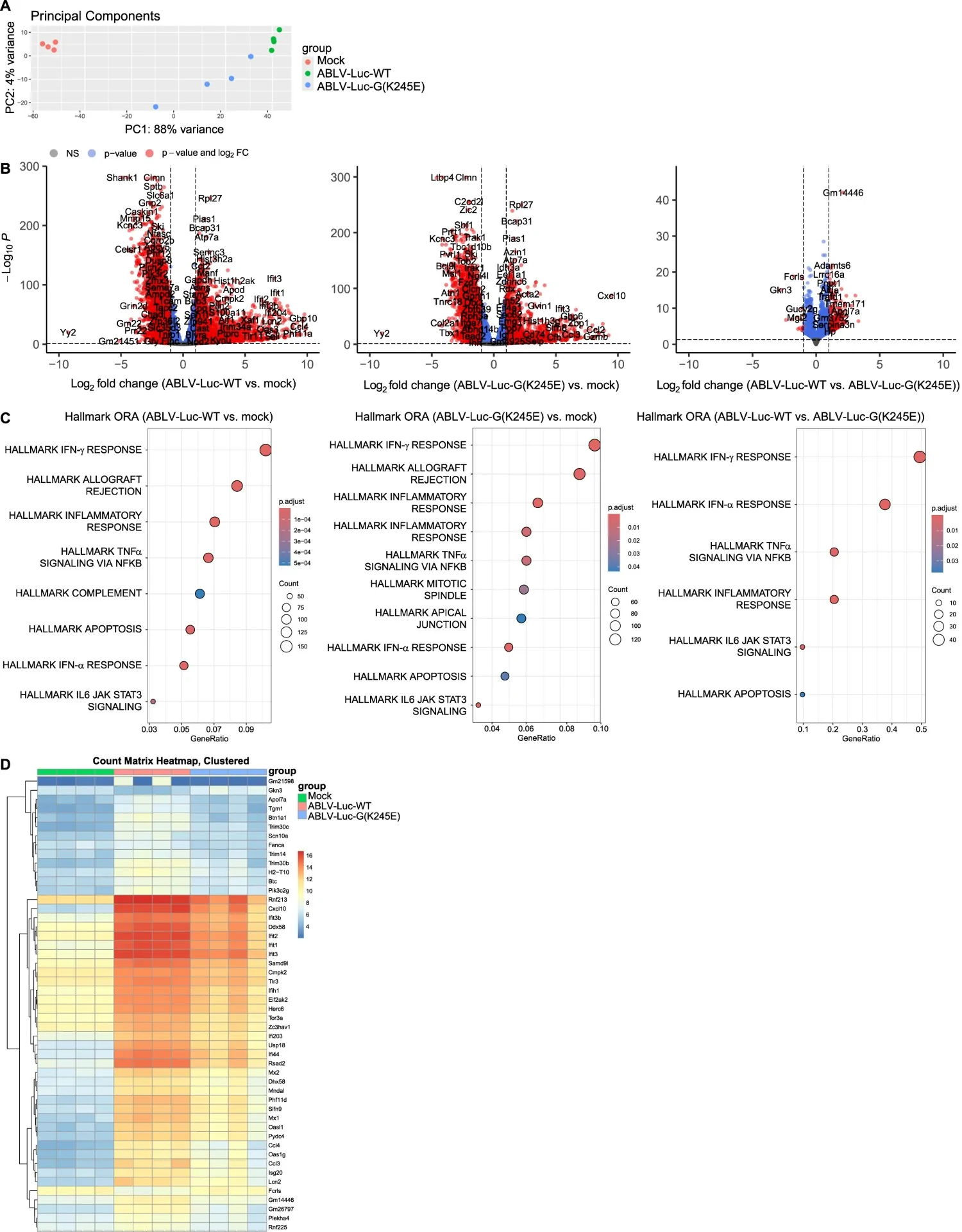
Highly similar inflammatory and antiviral responses are induced by ABLV-Luc-WT and ABLV-Luc-G(K245E). (A to D) (*n* = 4/group; brains collected at 14 days post-infection) (A) Principal component analysis of whole-brain RNA-seq profiles showing clustering of ABLV-WT and ABLV-Luc-G(K245E) infected samples and distinct separation of mock-infected samples. (B) Volcano plots of DEGs for the comparisons of ABLV + WT vs. mock-infected, ABLV-Luc-G(K245E) vs. mock-infected, and ABLV + WT vs. ABLV-Luc-G(K245E). Significant DEGs (*p* *<* 0.05) with a log_2_foldchange > 1 are highlighted in red and labelled with gene symbols. Genes below the log_2_foldchange cutoff of 1 are coloured in blue. (C) GSEA dot plots showing pathway enrichment for the comparisons ABLV + Luc-WT vs. mock-infected, ABLV-Luc-G(K245E) vs. mock-infected, and ABLV + WT vs. ABLV-Luc-G(K245E). Dot colour indicates the adjusted *p*-value for each enriched pathway, and dot size represents the number of genes contributing to the enrichment within each gene set. (D) Clustered heatmap of the top 50 DEGs from rlog-transformed RNA-seq counts. DEGs were filtered for immune relevance using msigdbr collections. Genes were further selected for a log_2_ fold-change >1 and base mean >80. Colour intensity indicates relative normalized expression across all samples.

Gene set enrichment analysis identified strong enrichment of immune and inflammatory pathways in mice infected with both ABLV-Luc-WT and ABLV-Luc-G(K245E) relative to mock-infected controls, with a high degree of overlap in pathways identified. Comparing ABLV-Luc-WT with ABLV-Luc-G(K245E) revealed a significantly higher enrichment in interferon and inflammatory response pathways in mice infected with ABLV-WT-Luc (Figure 7(C)). Hierarchical clustering of the top 50 DEGs revealed strong inflammatory and antiviral gene signatures in both the ABLV-Luc-WT and ABLV-Luc-G(K245E) groups relative to mock-infected controls. However, despite these overall similarities, comparison of ABLV-Luc-WT and ABLV-Luc-G(K245E) demonstrated a stronger inflammatory and antiviral transcriptomic signal in the ABLV-Luc-WT group (Figure 7(D)). Collectively, the data in Figure 7 demonstrate qualitatively similar gene expression signatures in the brain that are elicited by infection with the WT and G(K245E) viruses. Considering these data in concert with the longitudinal tracing of infections in Figure 6B,C, the simplest conclusions are that (i) the magnitude of inflammatory and antiviral gene expression is likely generally proportional to the viral load in the brain; and (ii) that inflammatory gene expression in the brain is unlikely to be a primary driver of morbidity and mortality due to lyssavirus infection.

## Discussion

In this study, we used structural and biochemical approaches to define a conserved epitope on Domain III (PHD) of the lyssavirus glycoprotein that is targeted by the broadly neutralizing antibodies A6 and F11. Key PHD residues contacted by A6 and F11 are highly conserved within phylogroup I, explaining the ability of both mAbs to neutralize across lyssavirus strains in this phylogroup [19]. By contrast, substitutions in this PHD region in lyssaviruses outside of phylogroup I likely disrupt critical hydrogen bonds, salt bridges, and hydrophobic interactions, accounting for the inability of F11 and A6 to neutralize phylogroup II and ungrouped lyssaviruses. These results are in contrast to the distinct PHD region bound by RVC20, which is more broadly conserved across lyssaviruses, enabling RVC20 to neutralize some viruses outside of phylogroup I [22]. The phylogroup-restricted nature of the A6/F11 epitope suggests that additional engineering, such as epitope scaffolding or multivalent presentation, may be required to achieve broader protection that encompasses all lyssaviruses.

The A6/F11-binding site represents a previously underappreciated region of vulnerability in the lyssavirus glycoprotein, distinct from other antigenic sites such as the RVC20-binding epitope. The ability of A6 and RVC20 to simultaneously engage non-overlapping epitopes on the PHD highlights the structural accessibility and functional relevance of this domain. Importantly, A6 binding compatibility with the prefusion conformation of RABV G indicates that this epitope is exposed on the native viral surface. The observed incompatibility of A6 binding to the postfusion conformation of G strongly suggests that these antibodies neutralize by stabilizing the prefusion state, thereby blocking the conformational changes required for membrane fusion. Although we were unable to directly demonstrate incompatibility with the postfusion conformation of G, due to technical limitations (e.g. the metastable nature of G, which readily switches between pre- and postfusion conformations), in silico structural alignment of antibody-bound complexes with prefusion and postfusion conformations is a well-established and generally accepted approach (e.g. [65,66]). Notably, our structural characterization of A6 places its binding site proximal to antigenic site II of the lyssavirus glycoprotein. While we utilized RVC20 as a comparator due to its well-characterized human origin, distinct epitope, and compatibility for ternary complex formation, future comparative studies with additional human or humanized mAbs targeting antigenic site II (e.g. humanized 1112-1 [58]) would further elucidate the relationship between epitope specificity and therapeutic efficacy.

Notably, similar therapeutically effective mAbs to the Nipah virus and Hendra virus fusion (F) glycoprotein also bind to F, locking it in the prefusion conformation and preventing infection of target cells [67,68]. Moreover, the A6-PHD-RVC20 structure demonstrates that A6 and RVC20 can bind the PHD simultaneously without steric interference, supporting the feasibility of antibody combinations targeting distinct PHD epitopes. Such combinations may enhance neutralization potency and mitigate viral escape by imposing multiple constraints on the glycoprotein conformation. Collectively, our structural data show that the targeting of conserved epitopes within Domain III offers a promising strategy for focused design of broadly effective therapeutic mAbs and vaccines.

Our results with A6 as a therapy for CNS-resident lyssavirus infection are very similar to our prior observations with F11 [18], although the efficiency of rescue from mortality via therapy at day 7 post-infection was superior with F11 (83% survival, vs 33% with A6). In comparing A6 and RVC20, although these mAbs had similarly potent *in vitro* neutralization potency, the kinetic profiles of *in vivo* efficacy were distinct, with A6 exhibiting maximal potency in rescue of animals from mortality on day 5 post-infection (100% survival), similar to F11 [18]. By contrast, the maximal rescue from mortality by RVC20 was on day 7 post-infection (100% survival), with day 5 administration being less effective (50% survival); also, survival following day 9 administration of RVC20 was superior to A6 (67% survival with RVC20, vs. 17% survival with A6). Interestingly, we noted a trend toward better functional recovery in the A6-treated animals, with 8 of 9 of the surviving treated animals returning to a disease score of 0 and recovering an ability to complete at least 30 s on the inverted mesh test, vs. only 5 of 13 and 6 of 13 (respectively) surviving animals treated with RVC20 recovering these functional abilities to the same degree. Although neither the mesh inversion test nor treatment at day 9 post-infection were performed in our analyses of F11, the disease score results were similar to A6, with 6 of 6 surviving day 5-treated animals and 4 of 5 surviving day 7-treated animals returning to a disease score of 0 [18]. Further study will be required to better define the underlying mechanisms governing the distinct therapeutic kinetics and efficacy profiles of A6, F11 and RVC20.

We also tested the ability of these therapeutic mAbs to neutralize Ken-1, a currently circulating strain of wild canine RABV. All three mAbs were quite potent *in vitro*, although the IC_50_ of A6 and F11 was approximately 3-fold higher than RVC20. With regard to *in vivo* therapeutic efficacy, A6 rescued 100% of animals from Ken-1-induced mortality when administered at day 5 post-infection. Thus, A6 is effective in a pre-clinical setting for the treatment of a currently circulating canine RABV isolate, and we expect the same would also be true of F11 and RVC20. Moreover, given the broad *in vitro* efficacy of these mAbs against phylogroup I lyssaviruses [19,22], the expectation is that highly effective rescue from mortality would extend to many other currently circulating RABV strains. However, although our data demonstrate robust efficacy against the Ken-1 RABV isolate, future *in vivo* studies evaluating A6 against additional geographically diverse RABV strains will further establish the breadth of its therapeutic potential.

Transcriptomics analyses of RNA from brains of control animals and ABLV-infected animals treated with either A6 or control mAb m102.4 demonstrated that ABLV-infection is associated with a substantial inflammatory and antiviral response when animals were given the control mAb. These results are consistent with what has been reported in the literature for both murine and human RABV infections [69,70]. Although a strong inflammatory response has been reported to be a characteristic of murine lyssavirus infections (and not human infections) [71], other studies have shown that inflammatory cytokines, including IFNγ and IL1β, are more pronounced in the paralytic form of rabies (the type of rabies in this mouse model) in non-rodent species including dogs; additionally, the absence of inflammatory responses reported in human post-mortem brains may reflect the late stage of disease in patients from whom tissues were harvested, rather than providing evidence of a reduced or absent manifestation of rabies-associated inflammation in humans [72]. Notably, A6 therapy was highly effective at suppressing inflammatory and antiviral gene expression induced by ABLV infection, consistent with the rescue of 100% of infected animals treated at day 5. Given our previous demonstration of a CD4 T cell-enriched leukocyte infiltration in F11-treated animals at this same post-infection time point [18], we hypothesize that these blood-derived leukocytes play a key role in suppressing both the viral infection and the inflammatory response to that infection. However, future studies will be necessary to provide evidence to support this hypothesis, as well as a mechanistic connection between these postulated activities and mAb therapy.

Comparative gene expression analysis of RNAs from the brains of mice infected with wild-type ABLV-Luc and the attenuated strain, ABLV-Luc-G(K245E) revealed that both viruses induce a highly similar inflammatory and antiviral response, despite the fact that infection with the attenuated strain is non-lethal. The attenuated virus experiment was critical to dissociate inflammation from other known and unknown consequences of lyssavirus infection as determinants of mortality. Despite similar inflammatory gene signatures, mice infected with ABLV-Luc-G(K245E) survived while WT-infected mice succumbed. This demonstrates that neuroinflammation, while present, is not a sufficient predictor of lethal outcome and supports our conclusion that the mechanism of the A6 therapeutic effect extends beyond simple suppression of inflammatory responses. Moreover, the broad and robust induction of inflammatory genes in the brain may mask gene expression signatures that are mechanistically linked to animal mortality, making the process of identifying A6-dependent gene expression changes that are relevant to survival more challenging. Further studies will be required to define the precise mechanisms by which peripherally administered mAbs can rescue animals from death. Other approaches, such as single-cell analyses of gene expression in infiltrating leukocytes, may provide mechanistic insights into specific programs of gene expression that are linked to the effects of A6 that lead to protection from mortality and functional recovery.

Overall, this work has provided further mechanistic insights regarding molecular mechanisms and gene expression alterations that are connected to the therapeutic response of animals to single-dose peripheral mAb therapy. Specifically, we have identified a binding site on G and a mechanism for viral neutralization for A6 and F11. We have also shown that peripherally administered single-dose therapy with A6 and RVC20 leads to survival and substantial functional recovery for all or some animals, even when administered as late as day 9 post-infection. These preclinical data thus build on previous studies from us [18] and others [21,73], suggesting that clinical development of a mAb-based therapy for symptomatic human rabies may be feasible.

## Supplementary Material

Supplemental Figure 3 (PHD-A6-RVC20 ternary complex).eps

Supplemental Figure 9 (serum cytokines).eps

Table S1 (FINAL).docx

Supplemental Figure 1 (Design of ABLV PHD).eps

Supplemental Figure 8 (H&E stains - histology).eps

Supplemental Figure 6 (Ken-1 dose response).eps

Supplemental Figure 5 (ABLV-luc + RVC20).eps

Supplemental Figure 2 (Negative stain EM - ABLV PHD).eps

Supplemental Figure 7 (ABLV-luc + m102.4 or A6 - mouse data for transcriptomics experiment).eps

Supplemental Figure 4 (ABLV neutralization titrations).eps
