# Supplementary material for: Peripheral human mAb therapy yields modulation of neuroinflammation and long-term functional recovery from lyssavirus infection
Source: Emerg Microbes Infect. 2026 Sep 18;15(1):2736987. doi: 10.1080/22221751.2026.2736987 (PMC13637774; doi:10.1080/22221751.2026.2736987)
Supplement: Supplementary file 1 [file TEMI_A_2736987_SM2010.docx]

| **Table S1: Data Collection and Refinement Statistics for cryo-EM** | | | | |
| --- | --- | --- | --- | --- |
|  |  |  | |  |
|  | **ABLV-PHD:A6:RVC20** |  | |  |
|  | **EMD-79039** |  | |  |
|  | **PDB-38PM** |  | |  |
| **Data Collection** |  |  | |  |
| Microscope | FEI Titan Krios |  | |  |
| Voltage (kV) | 300 |  | |  |
| Electron dose (e-/Å²) | 50 |  | |  |
| Detector | Gatan K3 |  | |  |
| Pixel Size (Å) | 0.852 |  | |  |
| Defocus Range (µm) | -1.0 to -2.5 |  | |  |
| Magnification | 81,000 |  | |  |
|  |  |  | |  |
| **Reconstruction** |  |  | |  |
| Software | cryoSPARC |  | |  |
| Particles | 312,665 |  | |  |
| Symmetry | C1 |  | |  |
| Box size (pix) | 360 |  | |  |
| Resolution (Å) (FSC_0.143_)^&^ | 3.6 |  | |  |
|  |  |  | |  |
| **Refinement (Phenix)^#^** |  |  | |  |
| Protein residues | 557 |  | |  |
| Chimera CC | 0.83 |  | |  |
| Resolution (Å) (FSC_0.5_) | 3.8 |  | |  |
| EMRinger Score | 2.71 |  | |  |
| R.m.s. deviations |  |  | |  |
| Bond lengths (Å) | 0.003 |  | |  |
| Bond angles (°) | 0.554 |  | |  |
|  |  |  | |  |
| **Validation** |  |  | |  |
| Molprobity score | 2.66 |  | |  |
| Clash score | 12.19 |  | |  |
| Rotamers outliers (%) | 6.21 |  | |  |
| Ramachandran |  |  | |  |
| Favored regions (%) | 92.84 |  | |  |
| Disallowed regions (%) | 0 |  | |  |
|  |  | |  | |
| ^&^Resolutions are reported according to the FSC 0.143 gold-standard criterion. | | | | |
| ^#^Statistics are reported for the protein residues within the complex. | | |  | |
